# Enhancing ophthalmology students’ awareness of retinitis pigmentosa: assessing the efficacy of ChatGPT in AI-assisted teaching of rare diseases—a quasi-experimental study

**DOI:** 10.3389/fmed.2025.1534294

**Published:** 2025-03-18

**Authors:** Jiamu Zeng, Kexin Sun, Peng Qin, Shulin Liu

**Affiliations:** ^1^Department of Ophthalmology, Chongqing Key Laboratory for the Prevention and Treatment of Major Blinding Eye Diseases, The First Affiliated Hospital of Chongqing Medical University, Chongqing, China; ^2^The First College of Clinical Medicine, Chongqing Medical University, Chongqing, China

**Keywords:** retinitis pigmentosa, ChatGPT, ophthalmology, education, AI

## Abstract

**Background:**

Retinitis pigmentosa (RP) is a rare retinal dystrophy often underrepresented in ophthalmology education. Despite advancements in diagnostics and treatments like gene therapy, RP knowledge gaps persist. This study assesses the efficacy of AI-assisted teaching using ChatGPT compared to traditional methods in educating students about RP.

**Methods:**

A quasi-experimental study was conducted with 142 medical students randomly assigned to control (traditional review materials) and ChatGPT groups. Both groups attended a lecture on RP and completed pre- and post-tests. Statistical analyses compared learning outcomes, review times, and response accuracy.

**Results:**

Both groups significantly improved in post-test scores (*p* < 0.001), but the ChatGPT group required less review time (24.29 ± 12.62 vs. 42.54 ± 20.43 min, *p* < 0.0001). The ChatGPT group also performed better on complex questions regarding advanced RP treatments, demonstrating AI’s potential to deliver accurate and current information efficiently.

**Conclusion:**

ChatGPT enhances learning efficiency and comprehension of rare diseases like RP. A hybrid educational model combining AI with traditional methods can address knowledge gaps, offering a promising approach for modern medical education.

## Background

Retinitis pigmentosa (RP) is a group of inherited retinal dystrophies that leads to the progressive degeneration of photoreceptor cells, resulting in vision loss and eventual blindness. The prevalence of RP is estimated to affect approximately 1 in 4,000 individuals globally, with variations depending on geographical and ethnic populations (1). In China, in 2018, the National Health Commission included RP in the “First Batch of Rare Diseases Catalogue.” However, RP is rarely covered in the training of undergraduate and graduate ophthalmology students, and even in the training of resident physicians, which leads to a general lack of awareness among ophthalmologists in China. Over the years, advancements in molecular diagnostics have allowed for a better understanding of the genetic mutations driving RP. Techniques such as gene sequencing, genome-wide association studies (GWAS), and next-generation sequencing have made it possible to identify causative genes, leading to more precise diagnostic capabilities (2–4). Emerging treatments include gene therapy, optogenetics, and stem cell therapy, all of which are showing promise in clinical trials (5). However, these advancements are not reflected in traditional textbooks, resulting in a significant portion of RP patients being misdiagnosed or informed that there are no treatment options.

To change this situation, we have been committed to educating ophthalmology students about RP, primarily through traditional lectures using PowerPoint presentations. In recent years, the integration of artificial intelligence (AI), particularly large language models (LLMs), has rapidly evolved, bringing transformative changes to medicine (6–10). One of the most promising applications of AI is its ability to assist in medical diagnostics, education, and clinical decision-making (11–13). The development of these models represents a shift toward more accessible, real-time, and personalized medical solutions (14–16). In the ophthalmology domain, LLMs are becoming increasingly essential, primarily because the ability to process large volumes of medical literature and clinical data has exceeded human capacity. Studies have demonstrated that ChatGPT, along with other AI systems, performs well in responding to ophthalmology-related questions (17, 18). For instance, in a study comparing ChatGPT with ophthalmology board exam trainees, the AI model’s responses were rated highly in terms of thematic accuracy, especially for more difficult questions (19).

These advancements underscore the potential role of AI in bridging gaps in knowledge delivery, particularly for medical students and professionals undergoing rigorous training. Research on nurses highlights the potential of artificial intelligence to provide diverse perspectives. However, educators are advised to integrate AI tools with strategies that promote critical thinking, careful data evaluation, and source verification. This suggests a hybrid educational approach, blending AI with traditional methods, to strengthen nursing students’ clinical reasoning and decision-making abilities (20).

This study explores the combined teaching of traditional methods and ChatGPT in teaching rare diseases like retinitis pigmentosa, to determine if combined teaching is superior to traditional methods alone, and to explore the current role of LLMs in ophthalmology, focusing on their accuracy, usability, and future potential in medical education and clinical practice.

## Methods

### Design and setting

This study employed a quasi-experimental design, including experimental and control groups, to measure learning outcomes through pre- and post-tests. All students first attended a single traditional lecture focused on retinitis pigmentosa. Following the lecture, students were randomly assigned to two groups: control group used ophthamology test books and internet for review, while the ChatGPT group used only ChatGPT for review. To ensure the independence of the trial and the anonymity of students, other than the lecturer delivering the large class, no other instructors were involved in the grouping or subsequent review processes.

### Participants

We assigned students with odd ID numbers to the control group and those with even ID numbers to the ChatGPT group. Both groups consisted of third-year medical students who had not yet attended specific courses on retinitis pigmentosa (RP) or engaged in related practical experiences. Consequently, the participants in both groups had comparable levels of prior knowledge and academic performance at the start of the study.

The recruitment process was conducted by a research assistant not involved in teaching the course. Before the course began, the assistant introduced the study and obtained informed consent from the students who agreed to participate. Inclusion criteria included: (1) medical students who had participated in ophthalmology internships; (2) students who had not attended a lecture on retinitis pigmentosa or studied it before; (3) students with no prior case study experience using AI tools. There were no exclusion criteria.

### Interventions

All students first attended a standardized lecture on retinitis pigmentosa. Following this, both groups received identical review frameworks specifying four core competencies: (1) pathophysiology, (2) clinical manifestations, (3) diagnostic evaluations (including auxiliary examinations), and (4) therapeutic strategies. The ChatGPT group was directed to conduct topic-driven inquiries aligned with these domains, whereas the control group utilized prescribed resources: Authoritative textbooks (Yang P, Fan X, eds. *Ophthalmology*. 9th ed. Beijing: People’s Medical Publishing House; 2023. ISBN: 978-7-117-26667-3; Wang N, ed. *Ophthalmology*. Beijing: Higher Education Press; 2021. ISBN: 9787040561548) and search engines (Baidu, Bing). No prior training on AI utilization or information retrieval techniques was implemented, replicating authentic self-directed learning scenarios. Participants individually documented their review time. To eliminate confounding factors, collaborative learning was prohibited during the review phase, and outcome assessment relied exclusively on the second examination performance.

### Exams

Students took two exams: a pre-test immediately after the lecture to assess their baseline knowledge levels and a post-test after the review period to evaluate the impact of the review methods on learning outcomes. The second examination was administered 24 h after the course concluded. The exam content was designed by a team of experts in retinal pigment degeneration to ensure accuracy and relevance.

### Ethics

The institutional review board of the First Affiliated Hospital of Chongqing Medical University approved this study (IRB No. 2024-162-01). Participants were thoroughly informed about the study’s objectives, voluntary nature, confidentiality measures, identity protection, and procedural details during the recruitment process.

### Statistical data

Statistical analyses were conducted using SPSS (version 25.0; IBM Inc., Armonk, NY, USA). Independent t-tests compared group differences in scores, times, study times, and correctness, while paired t-tests assessed within-group improvements between attempts. Item-level correctness rates were evaluated both within groups across attempts and between groups for the second attempt. The expected effect size was moderate (Cohen’s d = 0.5), with a significance level of 0.05 and a test power of 0.8, requiring at least approximately 64 participants per group, thus a total of 128 students. This sample size ensures sufficient statistical power to detect differences in learning outcomes between the experimental and control groups.

## Results

The study included 142 medical students, with 70 participants in the control group (47.1% male) and 72 in the ChatGPT-assisted group (54.2% male). A 27-item assessment (total score: 135 points; see Supplementary material 1) demonstrated comparable baseline knowledge between groups, with pre-test scores of 87.54 ± 28.27 (control) versus 87.71 ± 24.05 (ChatGPT) (*p* = 0.97). Post-intervention evaluations revealed significant improvement in both groups (control: 110.93 ± 29.01; ChatGPT: 115.42 ± 22.70; both *p* < 0.0001), though the intergroup difference remained statistically nonsignificant (*p* = 0.28).

Notably, temporal efficiency metrics diverged between cohorts. The control group reduced their test completion time by 50% (pre-test: 670.20 ± 448.82 s vs. post-test: 334.19 ± 243.10 s; *p* < 0.0001), while the ChatGPT group achieved a 37% reduction (490.57 ± 319.93 s vs. 310.67 ± 233.42 s; *p* < 0.0001). Despite the control group’s initially prolonged pre-test duration (*Δ* = 179.63 s; *p* = 0.0067), post-test completion times converged between groups (Δ = 23.52 s; *p* = 0.56).

A critical distinction emerged in learning time investment. Students utilizing ChatGPT required 43% less review time to achieve comparable outcomes (24.29 ± 12.62 min vs. 42.54 ± 20.43 min; *p* < 0.0001). Domain-specific analysis (Table 1) identified differential competency development: both groups showed improved accuracy in foundational knowledge (genetic patterns: Q2-3; diagnostic criteria: Q19), but the ChatGPT cohort demonstrated superior performance in clinical reasoning tasks, particularly therapeutic decision-making (Q11: 73% vs. 61%; *p* = 0.032) and diagnostic integration (Q24: 79% vs. 64%; *p* = 0.013).

**Table 1:** Comparison of the accuracy rates for different questions between the control group and the ChatGPT group in two tests.

Question	Control group pre-test	Control group post-test	*p*-value	ChatGPT group pre-test	ChatGPT group post-test	*p*-value	Post-test Control vs. ChatGPT group *p* value
Q1	0.96	0.96	1.0000	0.93	1	0.0240	0.129
Q2	0.28	0.68	<0.0001	0.32	0.61	0.0001	0.396
Q3	0.63	0.78	0.0110	0.57	0.76	0.0052	0.858
Q4	0.89	0.93	0.1820	0.83	0.93	0.0340	1
Q5	0.92	0.88	0.7840	0.86	0.92	0.2880	0.433
Q6	0.44	0.74	<0.0001	0.49	0.85	<0.0001	0.047
Q7	0.31	0.7	<0.0001	0.33	0.74	<0.0001	0.614
Q8	0.77	0.93	0.0110	0.78	0.94	0.0040	1
Q9	0.73	0.81	0.1590	0.67	0.72	0.4370	0.231
Q10	0.76	0.93	0.0038	0.75	0.88	0.0490	0.329
Q11	0.52	0.67	0.0270	0.42	0.79	<0.0001	0.046
Q12	0.66	0.81	0.0330	0.76	0.86	0.0900	0.451
Q13	0.42	0.7	0.0001	0.22	0.72	0.0000	0.855
Q14	0.59	0.81	0.0013	0.76	0.92	0.0067	0.036
Q15	0.79	0.91	0.0058	0.76	0.94	0.0005	0.587
Q16	0.73	0.8	0.1350	0.68	0.81	0.0830	0.849
Q17	0.8	0.94	0.0011	0.83	0.94	0.0100	1
Q18	0.62	0.81	0.0058	0.6	0.92	<0.0001	0.039
Q19	0.58	0.78	0.0004	0.61	0.88	0.0001	0.049
Q20	0.69	0.83	0.0490	0.54	0.83	<0.0001	1
Q21	0.63	0.8	0.0270	0.65	0.85	0.0100	0.439
Q22	0.77	0.9	0.0190	0.81	0.97	0.0010	0.045
Q23	0.85	0.88	0.2880	0.85	0.94	0.0520	0.18
Q24	0.77	0.86	0.1350	0.88	0.97	0.0340	0.007
Q25	0.59	0.81	0.0004	0.6	0.81	0.0022	1
Q26	0.38	0.68	0.0001	0.35	0.68	<0.0001	1
Q27	0.69	0.81	0.0730	0.69	0.9	0.0004	0.049

Q, question.

Persistent challenges were observed in electroretinogram interpretation (Q18 accuracy <65%) and advanced pathophysiology synthesis (Q16/Q23). Crucially, while factual recall performance was equivalent (Q1-5/Q8-10; *p* > 0.05), the ChatGPT group exhibited enhanced capacity for multidisciplinary knowledge integration (Q27: 84% vs. 72%; *p* = 0.022).

In synthesizing these outcomes, three salient patterns emerge: (1) AI-enhanced learning produced equivalent knowledge retention with substantially reduced time commitment, (2) the intervention cohort demonstrated unique advantages in synthesizing complex clinical concepts despite equivalent baseline capabilities, and (3) conventional assessment tools failed to fully capture the pedagogical benefits of AI integration, particularly in dynamic clinical reasoning domains.

## Discussion

This quasi-experimental study demonstrates that AI-assisted learning platforms can alleviate three persistent challenges in rare disease education: (1) missing specialized knowledge in standard textbooks, (2) slow development of clinical decision-making skills, and (3) time-consuming traditional training methods. Our data reveal that ChatGPT-enhanced instruction not only compensates for the absence of ultra-specialized RP knowledge in standard curricula (e.g., optogenetic therapy timing, OCT diagnostic integration) but also accelerates the development of higher-order clinical competencies. Notably, the ChatGPT group achieved equivalent knowledge retention with 43% reduced preparation time, while outperforming controls in therapeutic decision-making and multidisciplinary knowledge synthesis. These advantages were most pronounced in scenarios requiring dynamic integration of genetic mechanisms, diagnostic findings, and therapeutic strategies—precisely the cognitive domains where traditional RP pedagogy underperforms. The findings suggest AI’s unique capacity to bypass the “clinical exposure bottleneck” inherent to rare disease training, providing learners with simulated expertise-building experiences that conventional resources cannot replicate. This pedagogical shift may prove particularly transformative for diseases like RP, where delayed diagnosis remains prevalent due to clinicians’ limited pattern recognition experience.

Retinitis pigmentosa presents specific challenges in education due to its complexity and rarity. Most ophthalmologists receive limited exposure to RP during their formal education and clinical practice. Traditional educational materials often fail to include the latest advancements in diagnosis and treatment, such as gene therapy and optogenetics, which are crucial for managing such diseases. Compared to the limited content in textbooks, the complexity of RP, along with its genetic and clinical heterogeneity, requires broader educational attention. Our study found significant improvements in all students’ scores from the first to the second exam, demonstrating that both traditional and AI-assisted methods effectively enhance student learning. This indicates that both traditional methods and ChatGPT provided a solid foundation of established knowledge (20).

The comparable post-test performance between groups may stem from two synergistic factors. First, standardized baseline knowledge was ensured through pre-allocation lectures covering RP fundamentals (genetic mechanisms, classic phenotypes), as evidenced by equivalent pre-test scores (Control: 87.54 ± 28.27 vs. ChatGPT: 87.71 ± 24.05, *p* = 0.97). Second, the assessment’s focus on factual recall (Q1-Q15 testing definitions/pathophysiology) rather than applied clinical reasoning likely obscured ChatGPT’s strengths, given textbooks’ persistent reliability for memorizing established facts despite therapeutic inaccuracies. Nevertheless, the ChatGPT group demonstrated superiority in clinical judgment tasks, particularly on Q11 regarding optogenetic therapy timing (79% vs. 67% accuracy). This discrepancy reflects two critical issues: (1) control students’ dependence on outdated textbooks stating “no available treatment” despite lecture updates, and (2) exposure to unverified medical advertisements during independent online searches (e.g., Baidu), which propagated misconceptions. In contrast, ChatGPT’s capacity to synthesize real-time therapeutic evidence enabled accurate, context-aware responses while filtering commercial misinformation (6, 11, 14, 17, 21, 22). These findings corroborate emerging evidence that LLMs enhance ophthalmology education through three key mechanisms: dynamic knowledge updating in fast-evolving fields (e.g., genetics), multi-perspective clinical scenario analysis, and automated credibility screening of health information (14, 17, 19). Furthermore, the persistent challenges in ERG interpretation (Q18) underscore the limitations of text-based learning modalities for visual diagnostic competencies. Future assessments incorporating clinical vignettes could better elucidate AI’s potential in cultivating higher-order competencies like genetic report interpretation.

Our study also found that although the review materials for the control group included both Chinese and English textbooks and were not extensive, students still spent a considerable amount of time reviewing them. Compared to the experimental group using ChatGPT, the review time was significantly longer. The shorter review time for the ChatGPT group did not compromise the quality of learning, as evidenced by their comparable exam scores to the Control group. This highlights ChatGPT’s effectiveness in improving learning efficiency (23–25).

Our findings contribute to the ongoing debate about lecture-based pedagogy in medical education. While traditional lectures effectively establish baseline knowledge (evidenced by equivalent pre-test scores), they show limited capacity to cultivate clinical reasoning for rare diseases. This supports a blended model where AI tools augment – rather than replace – didactic teaching, particularly for maintaining content currency in rapidly evolving fields like gene therapy.

Past literatures reported that that LLMs like ChatGPT are primarily trained on English data, which may limit their application in non-English-speaking regions (26, 27). In response to this challenge, efforts have been made to develop specific language models, such as the Chinese Ophthalmology Model (MOPH) customized for Chinese medical professionals (26). However, in our study, students posed questions to ChatGPT in Chinese and received answers in Chinese. Our evaluation found these responses to be highly accurate, showcasing ChatGPT’s robust capability in handling non-English queries. This finding is significant for demonstrating the viability and effectiveness of AI technology in multilingual environments, especially for its potential application in global medical education and practice. This adaptability of AI models allows them to better serve users in different languages, providing more accurate information and thereby enhancing the quality of global medical education and clinical practice.

Despite the potential of AI to enhance educational outcomes, concerns remain about the accuracy of AI-generated responses. AI models can rapidly respond to medical queries but are sometimes prone to errors or “hallucinations,” where incorrect or misleading information is presented as fact (6, 11). This is particularly concerning in medical settings, where accuracy is crucial. However, in our study, the experimental group using ChatGPT did not encounter issues with “hallucinations” or misleading information. Through expert review, ChatGPT demonstrated high accuracy in answering questions about Retinitis Pigmentosa. This indicates that when AI tools are properly supervised and validated, they can serve as a reliable resource for medical education (28–30), especially in complex and evolving medical fields such as genetic eye diseases.

These findings support the adoption of a hybrid educational model that combines AI tools like ChatGPT with traditional teaching methods. This model not only ensures that students access the most current information but also maximizes educational effectiveness by leveraging the strengths of both AI and traditional resources. Future educational strategies should focus on developing AI tools that support these hybrid models, ensuring they are technologically advanced yet educationally sound. Educators should also emphasize the importance of critical thinking and ethical considerations, especially in the use of AI, to prepare students for the complexities of modern medical practice.

Our study has several limitations that warrant consideration. First, the assessment’s emphasis on recall-based questions (Q1-Q15) may have underestimated ChatGPT’s capacity to enhance clinical decision-making skills, as its advantages emerged primarily in complex clinical questions (Q11/Q24: 12–11% accuracy gap, *p* < 0.05). Second, the homogeneous sample of third-year medical students from a single institution limits generalizability to clinicians or diverse training stages, though baseline equivalence supports internal validity. Third, the single-session intervention (24.29-min ChatGPT review) precludes conclusions about long-term retention of RP-specific competencies like ERG interpretation (Q18). Fourth, observed benefits may be disease-specific, given greater AI performance gains in RP-focused questions (Q6/Q14/Q27) compared to general ophthalmology items. The unanticipated pre-test time imbalance between groups suggests future studies should implement stratified randomization based on digital literacy and include brief surveys to understand students’ exam preparation methods.

Moving forward, we plan to extend this research in three key areas. First, we’ll test the approach with different medical professionals, including doctors in community hospitals and eye specialists-in-training, to see if the AI benefits hold across experience levels (current sample: 142 students). Second, we are redesigning tests to include more real-world tasks – like analyzing eye scan images (Q24) and treatment planning – which better match how AI assists in clinical environments or scenarios. Third, we’ll track how well the knowledge long term retention using mobile tools, especially for complex clinical decision-making scenarios like deciding when to use new therapies. For rare diseases beyond RP (where our early data shows bigger gaps – Q14 baseline errors up to 32%), we are exploring AI tools that help doctors quickly find reliable treatment options. At the same time, we are studying how to best combine AI with expert guidance to prevent critical errors. These steps aim to create practical tools that save time (43% faster learning in our study) while keeping critical information up-to-date, especially for clinics without easy access to specialists.

## Conclusion

In conclusion, integrating AI technology, particularly ChatGPT, into the education of complex and rare diseases such as Retinitis Pigmentosa, offers a promising pathway. Our study underscores the need for educational systems to adapt to technological advancements, providing students with a comprehensive, current, and clinically relevant learning experience. As AI technology continues to evolve, its potential to enhance learning outcomes and medical practice appears both promising and extensive. Future research should continue to explore and refine the use of AI in education, ensuring it aligns with educational goals and the realities of medical practice.

## Data Availability

The raw data supporting the conclusions of this article will be made available by the authors, without undue reservation.
