# Supplementary material for: Enhancing ophthalmology students’ awareness of retinitis pigmentosa: assessing the efficacy of ChatGPT in AI-assisted teaching of rare diseases—a quasi-experimental study
Source: Front Med (Lausanne). 2025 Mar 18;12:1534294. doi: 10.3389/fmed.2025.1534294 (PMC11959056; doi:10.3389/fmed.2025.1534294)
Supplement: Supplementary file 1 [file Table_1.DOCX]

Supplementary Material

# Supplementary Data

Test

1. Retinitis pigmentosa is primarily a: [Single-choice question]

A) Infectious disease

B) Genetic disorder (Correct answer)

C) Autoimmune disease

D) Metabolic disorder

2. The most common inheritance pattern of RP is: [Single-choice question]

A) Autosomal dominant inheritance

B) Autosomal recessive inheritance (Correct answer)

C) X-linked inheritance

D) Mitochondrial inheritance

3. The primary gene associated with X-linked RP is: [Single-choice question]

A) RHO

B) RPGR (Correct answer)

C) ABCA4

D) USH2A

4. Early symptoms of RP usually include: [Single-choice question]

A) Sudden vision loss

B) Night blindness (Correct answer)

C) Eye pain

D) Retinal hemorrhage

5. Which technique is most commonly used for diagnosing RP? [Single-choice question]

A) Optical coherence tomography (OCT)

B) Electroretinography (ERG) (Correct answer)

C) CT scan

D) MRI

6. The main pathological changes in RP are: [Single-choice question]

A) Macular edema

B) Dysfunction of cone cells

C) Degeneration of rod cells (Correct answer)

D) Retinal vascular abnormalities

7. When do RP patients typically start showing symptoms? [Single-choice question]

A) Childhood (Correct answer)

B) Adolescence

C) Middle age

D) Old age

8. The pathological mechanism of RP usually first affects: [Single-choice question]

A) Central vision

B) Peripheral visual field (Correct answer)

C) Color vision

D) Depth perception

9. Common imaging findings in RP patients include: [Single-choice question]

A) Optic nerve atrophy

B) Retinal pigment deposition (Correct answer)

C) Retinal hemorrhage

D) Cystoid macular edema

10. Gene therapy currently targets which type of RP patients? [Single-choice question]

A) Patients with high myopia

B) Patients with retinal detachment

C) Patients in the late stages of RP

D) Patients with specific genetic mutations (Correct answer)

11. At which stage of RP is optogenetic therapy applicable? [Single-choice question]

A) Early stage

B) End stage (Correct answer)

C) Middle stage

D) All stages

12. Electrophysiological examinations of RP patients typically show: [Single-choice question]

A) Completely normal results

B) Loss of photoreceptor function (Correct answer)

C) Delayed optic nerve conduction

D) Elevated intraocular pressure

13. Common complications of RP include: [Single-choice question]

A) Glaucoma

B) Cataracts (Correct answer)

C) Macular degeneration

D) Corneal opacity

14. Night blindness in RP is due to damage in which retinal cells? [Single-choice question]

A) Cone cells

B) Rod cells (Correct answer)

C) Retinal pigment epithelium

D) Optic nerve

15. Early symptoms of RP do not include: [Single-choice question]

A) Night blindness

B) Peripheral visual field loss

C) Retinal pigment deposition

D) Central vision loss (Correct answer)

16. Which current RP treatment has entered clinical trials? [Single-choice question]

A) Laser treatment

B) Gene replacement therapy (Correct answer)

C) Cataract surgery

D) Radiation therapy

17. Which is not an inheritance pattern of RP? [Single-choice question]

A) Autosomal dominant

B) Autosomal recessive

C) X-linked

D) Y-linked (Correct answer)

18. RP patients usually display which findings on an electroretinogram (ERG)? [Single-choice question]

A) Normal responses

B) Cone cell dysfunction

C) Rod cell dysfunction (Correct answer)

D) Optic nerve dysfunction

19. Typical fundus findings in RP patients include: [Single-choice question]

A) Macular edema

B) Pigment clumping

C) Optic disc atrophy

D) Bone spicule-like pigment (Correct answer)

20. Which test is most commonly used to assess visual field defects in RP patients? [Single-choice question]

A) OCT

B) Visual field testing (Correct answer)

C) Pupillary reflex

D) Intraocular pressure testing

21. The factor most closely related to the decline in visual function in RP patients is: [Single-choice question]

A) Reduced cone cell count

B) Rod cell dysfunction (Correct answer)

C) Abnormal optic nerve conduction

D) Lens opacity

22. Late symptoms of RP may include: [Single-choice question]

A) Complete vision loss (Correct answer)

B) Visual field defects only

C) Mildly blurred vision

D) Floaters

23. The main goal of gene therapy is: [Single-choice question]

A) Retinal reconstruction

B) Correcting mutated genes (Correct answer)

C) Reducing inflammation

D) Preventing neovascularization

24. In RP diagnosis, OCT is mainly used for: [Single-choice question]

A) Measuring intraocular pressure

B) Observing retinal layer structure (Correct answer)

C) Diagnosing glaucoma

D) Assessing the optic nerve head

25. RP patients often begin to experience visual damage in which area? [Single-choice question]

A) Peripheral visual field (Correct answer)

B) Central visual field

C) Night vision

D) Color vision

26. RP is often associated with which disease? [Single-choice question]

A) Glaucoma

B) Keratitis

C) Cataracts (Correct answer)

D) Optic nerve atrophy

27. The earliest affected cells in RP are: [Single-choice question]

A) Cone cells

B) Rod cells (Correct answer)

C) Retinal ganglion cells

D) Choroidal cells
